# Functional analysis of an intergenic non-coding sequence within *mce1 *operon of *M.tuberculosis*

**DOI:** 10.1186/1471-2180-10-128

**Published:** 2010-04-27

**Authors:** Monika Joon, Shipra Bhatia, Rashmi Pasricha, Mridula Bose, Vani Brahmachari

**Affiliations:** 1 Dr B R Ambedkar Centre for Biomedical Research, University of Delhi, Delhi-110007, India; 2 Vallabhbhai Patel Chest Institute, University of Delhi, Delhi-110007, India

## Abstract

**Background:**

The *mce *operons play an important role in the entry of *M. tuberculosis *into macrophages and non-phagocytic cells. Their non-redundant function as well as complex regulation is implied by the phenotype of *mce *mutants. Recently, *mce1 *operon was found to extend over 13 genes, *fadD5 *(Rv0166) being the first gene of the operon. The presence of a non-coding sequence of 200 base pairs between Rv0166 and Rv0167 is peculiar to *mce1 *among the four *mce *operons of *M.tuberculosis*. We have examined the function of this region.

**Results:**

We predicted putative promoter activity of the 200 base pairs of non-coding, intergenic region between Rv0166 and Rv0167 *in silico *using MEME software and designate it as intergenic promoter, IGPr. We demonstrate both promoter activity and a putative negative regulatory function of this fragment by reporter assays carried out in the surrogate host *M.smegmatis*. We find that the repressive elements not only control the native promoter but also repress a heterologous promoter of *M.smegmatis*. The higher activity of the intergenic promoter in a clinical isolate in comparison with the wild type sequence from *M.tuberculosis *H37Rv could be correlated with a point mutation within the negative element. We have mapped two transcription start sites for *mce1 *operon both of which are utilized in *M.tuberculosis *H37Rv as well as the clinical isolate VPCI591. Our studies show that the promoter activity in the non-coding region is relevant not only in reporter gene expression but also in the expression of *mce1 *operon in *M. tuberculosis *cells grown in synthetic medium.

**Conclusion:**

The *mce *operon of *M.tuberculosis *H37Rv potentially can be transcribed from two promoters P1 and P2, former mapping upstream of Rv0166 and the latter in the non-coding intergenic region between Rv0166 and Rv0167. The transcription initiation from P1 results in a transcript with Rv0166 while that from P2 will be without it. The sequences between the translation start site of Rv0167 and the promoter P2 have a negative regulatory role, as point mutation within the sequence leads to enhanced activity of P2 as well as a heterologous promoter from *M.smegmatis*. The mutation detected in the clinical isolate VPCI591 therefore behaves like a gain-of-function mutation.

## Background

Tuberculosis causes approximately two million deaths annually and it has been estimated that around two billion people are currently infected with the causative organism, *Mycobacterium tuberculosis *[1]. Attempts to understand the molecular basis of pathogenesis in tuberculosis include the analysis of genes involved in the entry of the bacillus following the initial identification of mammalian cell entry protein, Mce1A by Arruda *et al. *[2]. Subsequent whole genome analysis revealed the presence of four *mce *operons in *M.tuberculosis *H37Rv, consisting of eight genes with extensive similarity between each other [2,3]. Recently, Casali *et al. *[4] redefined the boundaries of *mce1 *making it an operon of 13 genes extending from Rv0166 to Rv0178. The importance of *mce *operons in virulence is illustrated by various phenotypes observed in knock-out strains and the expression profile of the operons in bacilli in culture and during infection [5-8].

The conservation of most of the *mce *operons in all members of the *Mycobacterium tuberculosis *complex, and the presence of orthologous *mce *genes throughout the genus Mycobacteria, including the non-pathogenic species *M.smegmatis *suggests their functional importance in processes besides pathogenicity [6,7,9-13]. Casali *et al. *[4] discovered that *fadD5 *gene (Rv0166) is also a part of the *mce1 *operon, adding to the probable functional diversity of *mce *operons.

In tune with the proposed functional diversity it has been suggested that *mce1 *operon could be under the control of a global stress regulator or multiple negative regulators [4,14]. Rv0165c, a homologue of GntR regulator of *mce1 *operon and Rv1963 a TetR family regulator of *mce3 *operon are characterized as negative regulators of the respective operons [4,14,15]. The poor consensus of the promoter sequence of *mce3 *operon at -10 and -35 positions is speculated to reflect the complex regulation of the operon and its ability to interact with multiple sigma factors [4]. Given the importance of *mce1 *operon and evidences from knock-out studies, any alteration in the expression or genetic polymorphism in *mce *operons would have significant consequence on the pathogenicity and the severity of infection [6-8,16,17].

Here we examine the function of the non-coding sequence between Rv0166 and Rv0167, which led us to detect both promoter and negative regulatory element within the sequence. A point mutation in the regulatory region abolishes the negative regulation resulting in enhanced promoter activity.

## Results

### Detection of a putative promoter in intergenic region of *mce1 *operon

**Figure 1:**
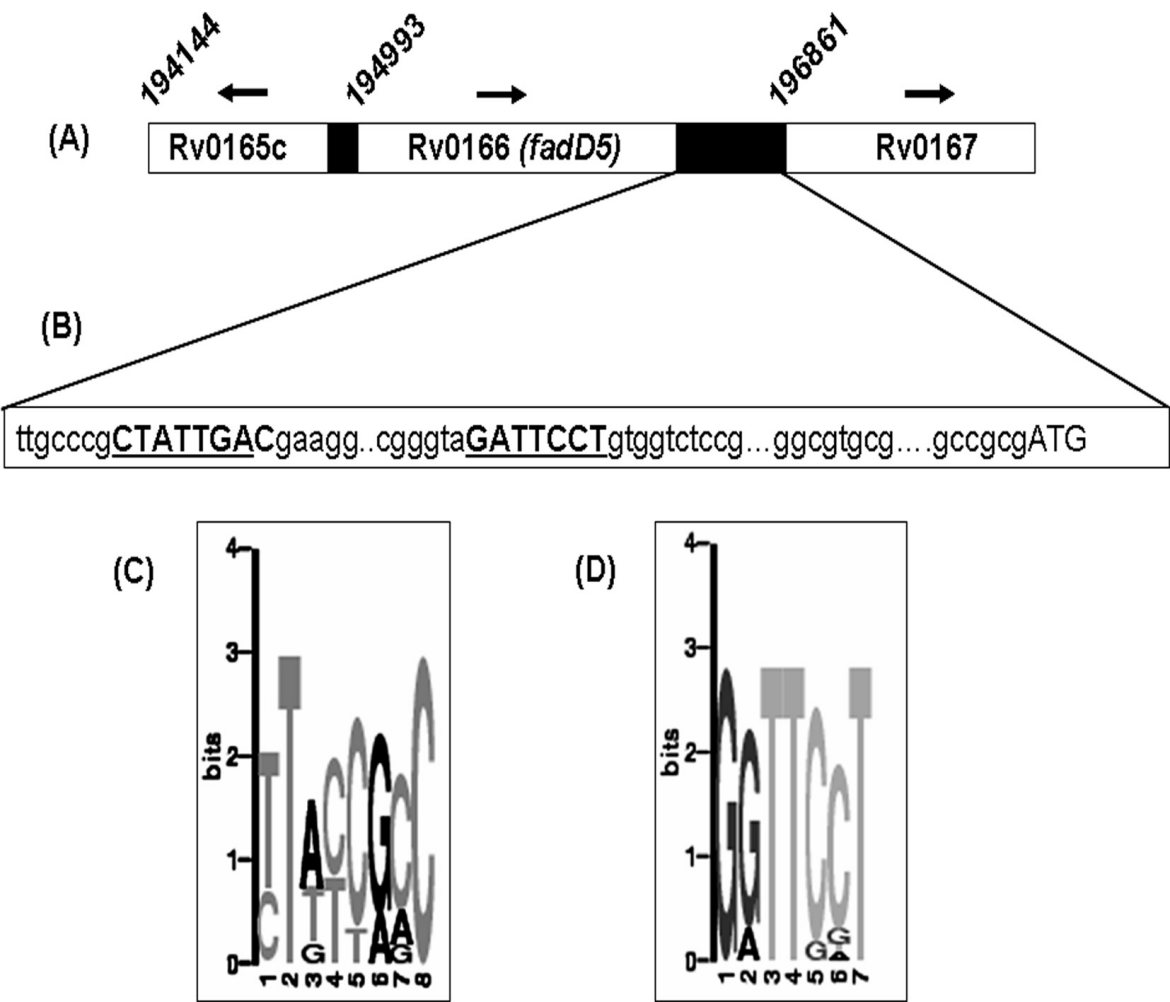
**Diagrammatic representation of intergenic region of *mce1 *operon**. (A)- Representation of the relative position of *mce1 *operon genes (within rectangles) in *M.tuberculosis*. Numbers above indicate the translational start site of the genes, arrows indicate the direction of transcription, filled bars indicate the intergenic regions. Figure is not drawn to scale. (B)- Mapping of the consensus motifs detected by MEME analysis of the predicted promoter sequences (IGPr). The motifs are highlighted in bold upper case. ATG is the translational start codon of Rv0167. (C, D)- Sequence logos of the two consensus sequences as given as the probability of occurrence at the given position with in the motif by the MEME software. The size of the letter indicating the strength of the consensus in the set of sequences analysed.

ORF analysis on sequences extending from Rv0166 (nucleotide 194993-196657) across Rv0167 (nucleotide 196861-197658) revealed the expected stop codon for Rv0166 at 196655 and the initiator codon for Rv0167 at 196861. However, no initiator codon was detected in the 200 base pairs between Rv0166 and Rv0167. This region therefore appears to be non-protein coding sequence within the *mce1 *operon in *M.tuberculosi*s H37Rv. We examined this sequence for probable promoter signature by *in silico *analysis. We retrieved 10 sequences with demonstrated promoter activity [18] in addition to the intergenic sequence of *mce1 *operon and aligned them with reference to the translational initiation site of the respective gene. The presence of consensus motif was analyzed using MEME http://meme.nbcr.net/meme3/meme.html. Two motifs GGTT [CG] [CG]T and TT [AT] [TC] [CT] [GA] [ACG]C were identified (p value > 1.31-e04) and both the motifs are present in the non-coding intergenic region between Rv0166 and Rv0167 of *mce1 *operon (Figure 1C &1D and Additional file 1). Since we detect landmarks of promoters known in *M.tuberculosis *within this region, we refer to it, henceforth as intergenic promoter (IGPr). We undertook the functional characterization of the predicted promoter activity of IGPr. We analyzed the effect of a point mutation in the IGPr, detected in a multi-drug resistant clinical isolate, VPCI591, under an independent analysis of genetic polymorphism in *mce *operons of clinical isolates of *M.tuberculosis *(unpublished).

### Promoter Activity of IGPr

**Figure 2:**
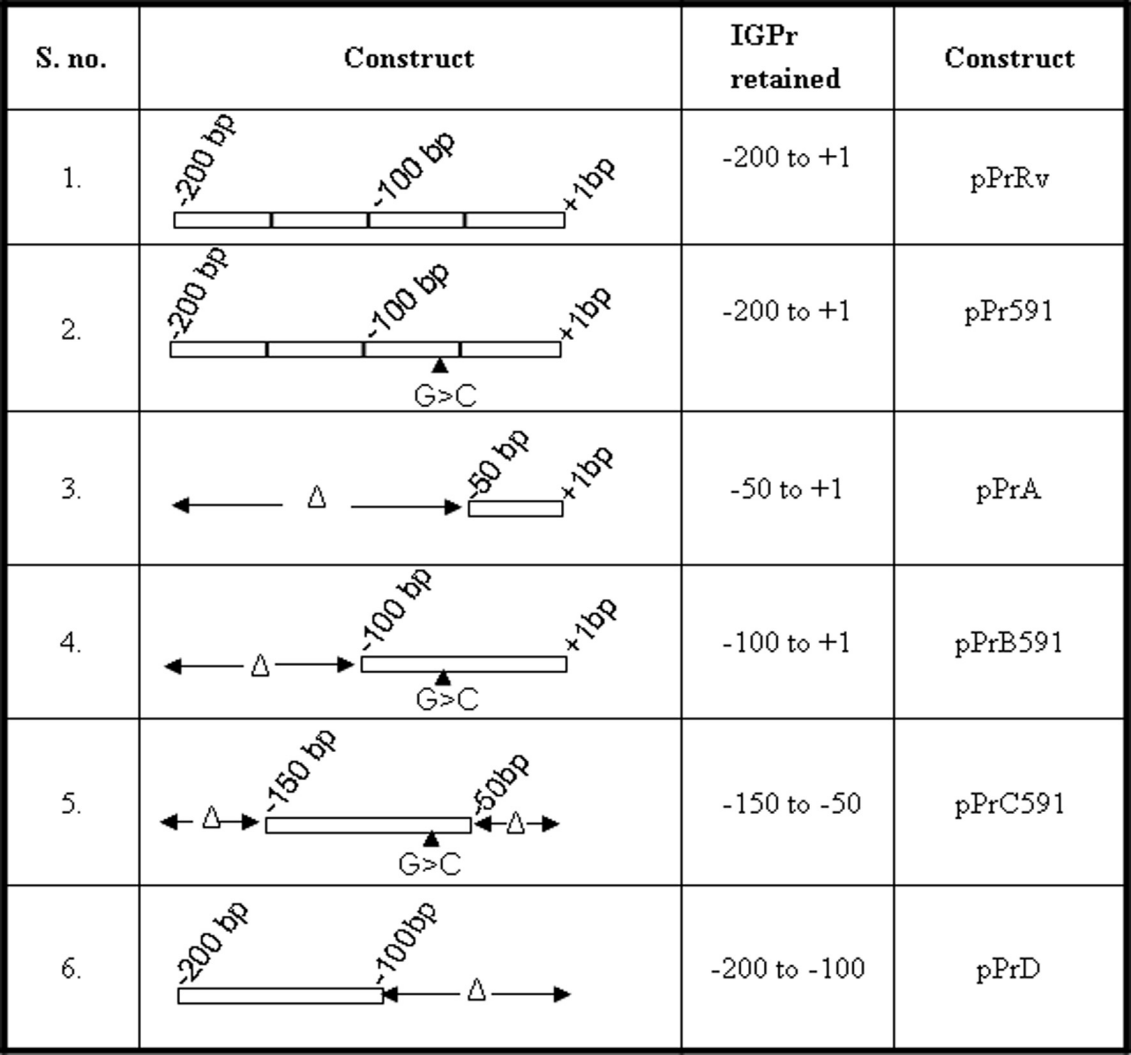
**Delineation of regulatory region**. Deletion constructs were generated to segregate promoter and the regulatory regions of IGPr. The column labeled as construct shows configuration of the inserts in different clones used in transformation of *M.smegmatis mc2 155*. The numbering is with reference to the translational initiation signal for Rv0167 as +1. The mutation in VPCI591 is shown as a filled triangle, the regions deleted in each clone is indicated by delta symbol. IGPr: 200 bp intergenic region between *Rv0166 *and Rv0167.

**Figure 3:**
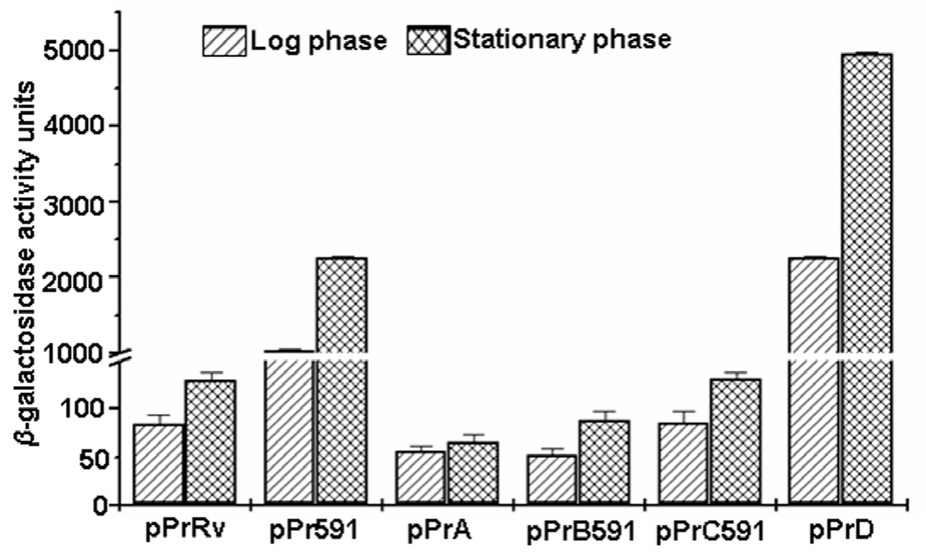
**Promoter Activity of IGPr deletion constructs**. *β*-galactosidase activity is expressed as nanomoles of ONPG converted to o-nitrophenol per min per mg of protein for the constructs. Each experiment was carried out in triplicates and standard deviation is indicated by error bars. The hatched and crossed bars represent log and stationary phase respectively. Please see Figure 2 for description of constructs used.

A 200 bp fragment containing IGPr sequence was amplified from *M.tuberculosis *H37Rv and cloned in promoter-less shuttle vector pSD5B, upstream of the *lacZ *as the reporter gene to generate pPrRv. Similarly 200 bp fragment from VPCI591 was cloned to produce pPr591 and both were tested for promoter activity in *M.smegmatis*. Different constructs used in the study are shown in Figure 2. Since a repression of *mce1 *operon at stationary phase was reported earlier [5], we analyzed the promoter activity of the two constructs both at log and stationary phase of growth, by ONPG assay using cell-free extracts from transformed *M.smegmatis *cells (Figure 3). The difference in the promoter activity of IGPr from VPCI591 (pPr591) is higher than that from *M.tuberculosis *H37Rv (pPrRv) by 12 fold (1025 vs 85 units of *β*-galactosidase activity) in log phase, which reaches 18 fold (2265 vs 130 units) in stationary phase (Figure 3). By comparing IGPr with previously characterized weak (pSD5WP) and strong (pSD5SP) promoters from *M.tuberculosis *[19], we find that pPrRv is a weak promoter while pPr591 acts as a strong promoter.

### Deletion analysis of IGPr region

In order to delineate the region of promoter activity within the 200 base pairs of IGPr, we made a series of deletion constructs. We generated amplicons corresponding to (-50 to +1), (-100 to +1), (-150 to-50) and (-200 to -100) and cloned them in pSD5B for expression in *M.smegmatis *(Figure 2). The promoter activity of 200 base pairs from *M.tuberculosis *H37Rv (pPrRv) is very low compared to that of the same region from VPCI591 (pPr591); 130 vs 2265 units respectively. The promoter activity is highest when -100 to +1 is deleted (pPrD) both in log (2255 units) and stationary phase of growth (4961 units, Figure 3); while it is negligible, when -200 to-100 is deleted (pPrB591; 52 and 89 units in log and stationary phase respectively). Additionally, the fragment containing only -150 to -100 (pPrC591) shows poor activity. Therefore we conclude that the promoter activity is restricted to around 50 base pairs from -200 to -150 within IGPr (Figure 3). Interestingly, significant promoter activity is detected in the construct that is deleted for -100 to +1 (pPrD). These results suggest that -100 to +1 region cloned in pPrRv has a negative effect which is lost in pPr591 derived from the clinical isolate VPCI591. We correlate this gain of expression due to loss of repression to the presence of a point mutation (G > C) at -61 in VPCI591.

**Figure 4:**
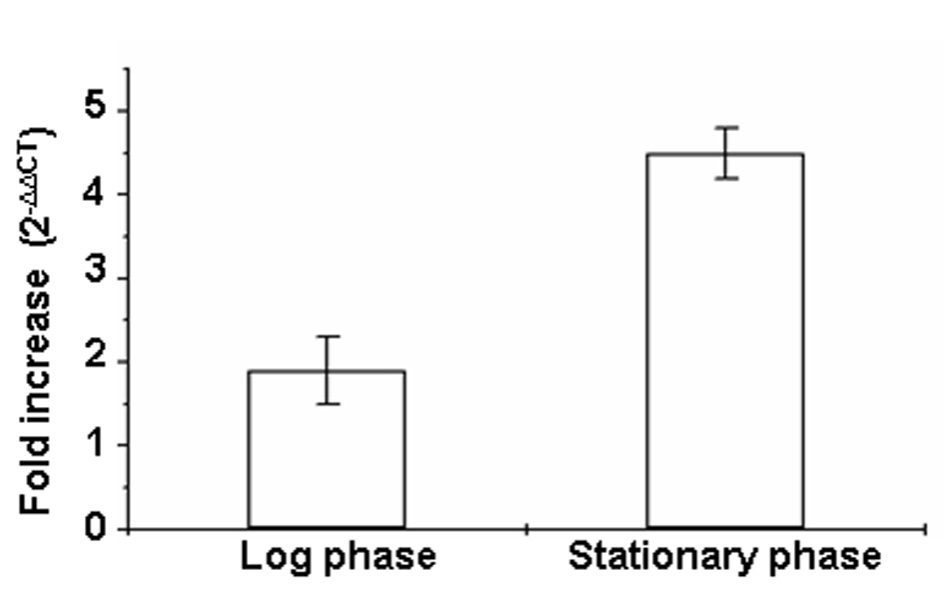
**Quantitative PCR analysis of *LacZ *reporter gene**. Fold difference in transcript level in pPr591 over that of pPrRv in log phase and stationary phase cultures are shown. The fold difference observed is the average of three independent experiments. Error bars represent the standard deviation.

To compare the mRNA levels from the two constructs, we isolated total RNA from *M.smegmatis *transformed with pPrRv (200 base pairs from *M.tuberculosis *H37Rv) and pPr591 (200 base pairs from VPCI591) and the transcript level was estimated by quantitative PCR with *lacZ *as target gene and *sigA *as the endogenous control in log and stationary phase. At log phase there is nearly two fold increase in *lacZ *transcripts in pPr591 as compared to pPrRv whereas in stationary phase it is more than four fold (Figure 4). The correlation between *β*-galactosidase activity and the mRNA levels of *lacZ *clearly indicate greater transcriptional activity in pPr591 than pPrRv. The difference in enzyme activity is much higher than the difference in mRNA levels as known in other cases [20-22].

### Mapping the transcription start site in *M.tuberculosis*

**Figure 5:**
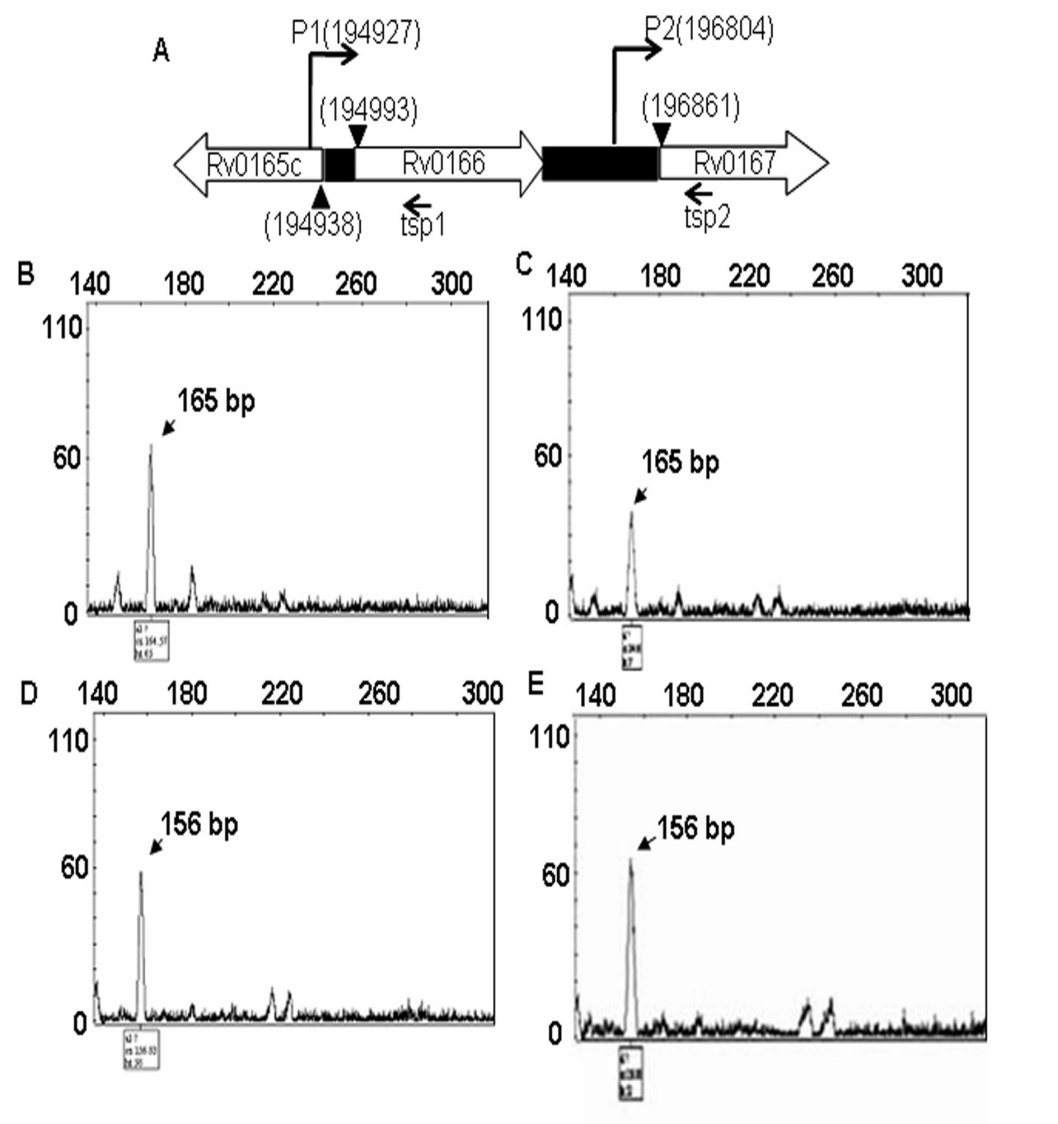
**Mapping of transcription start site (TSS) in *mce1 *operon**. A -Line diagram indicating the position of primers used for mapping TSS by primer extension. The numbers in parenthesis indicate the map position on the reference sequence of *M.tubersulosis *H37Rv. Filled boxes indicate non-coding regions, filled arrowheads indicate translation start site, tsp1 is HEX-labeled primer beginning at 195092, tsp2 is FAM-labeled primer beginning at 196960. P1 and P2 represent the TSS detected. B-E show Genescan analysis of the products of primer extension reactions on mRNA from *M.tuberculosis *H37Rv (B, D) and VPCI591 (C, E) with fluorescence labeled primers is shown in A. The peak at 165 bp position is transcript from P1 promoter and the peak at 156 position transcript from P2 promoter.

We identified transcription start site of Rv0166 and Rv0167 *in vivo *in *M.tuberculosis *H37Rv and VPCI591 using fluorescence tagged primers in primer extension assay using RNA templates. The absence of DNA contamination in RNA preparation was confirmed by PCR for Rv0166 and Rv0167 in absence of reverse transcriptase (data not shown). The sizing of the products was carried out by genescan analysis and the TSS was detected at -65 position from the translation initiation site of Rv0166 and at -56 position from the translation initiation site of Rv0167 (Figure 5B-E), suggesting that there are two potential promoters for *mce1 *operon generating two transcripts, one including Rv0166 and the other without it (Figure 5A). Further, this demonstrated that both promoters are active in the genomic context of *M.tuberculosis*. Considering the translation initiation site of Rv0167 as +1, we map the transcription start site within IGPr at -56 position and the mutation in VPCI591 at -61 position.

### Estimation of *mce1 *operon transcript levels in *M.tuberculosis*

**Figure 6:**
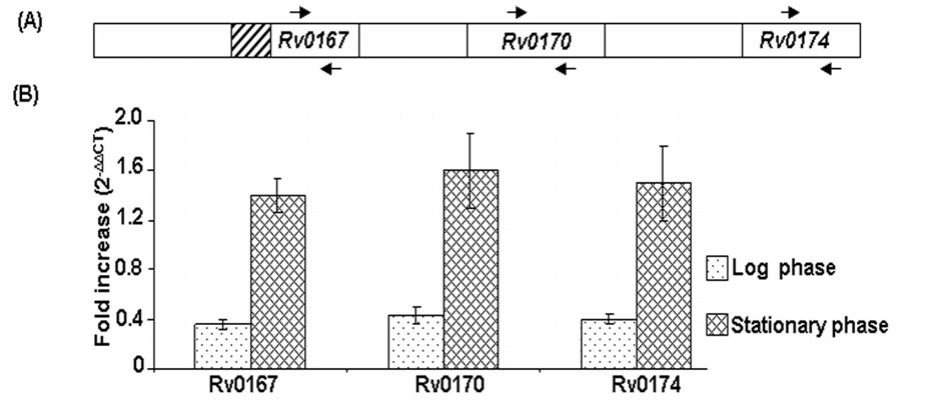
**Quantitative PCR analysis of *mce1 *operon in *M.tuberculosis *H37Rv and VPCI591**. (A)- Diagrammatic representation [not to scale] of the *mce1 *operon. Arrows indicate the position of primers. The hatched box depicts IGPr region. (B)- Fold difference in transcript level in VPCI591 over that of *M.tuberculosis *H37Rv for Rv0167, Rv0170 and Rv0174 in log phase (dotted) and stationary phase (hatched). The fold difference observed is the average of three independent experiments. Error bars represent the standard deviation.

The transcript level of Rv0167, Rv0170 and Rv0174 of *mce1 *operon downstream to IGPr in *M.tuberculosis *and VPCI591 was analyzed by quantitative PCR with *rpoB *as the endogenous control (Figure 6A). The data reveals 1.5 fold upregulation of the *mce1 *operon genes in VPCI591 as compared to *M.tuberculosis *H37Rv (Figure 6B). The difference at protein level is considerably higher than at the transcript levels in case of *β*-galactosidase, similar enhancement in Mce1 protein levels could also be anticipated.

### Effect of the regulatory sequence of IGPr on heterologous promoter

**Figure 7:**
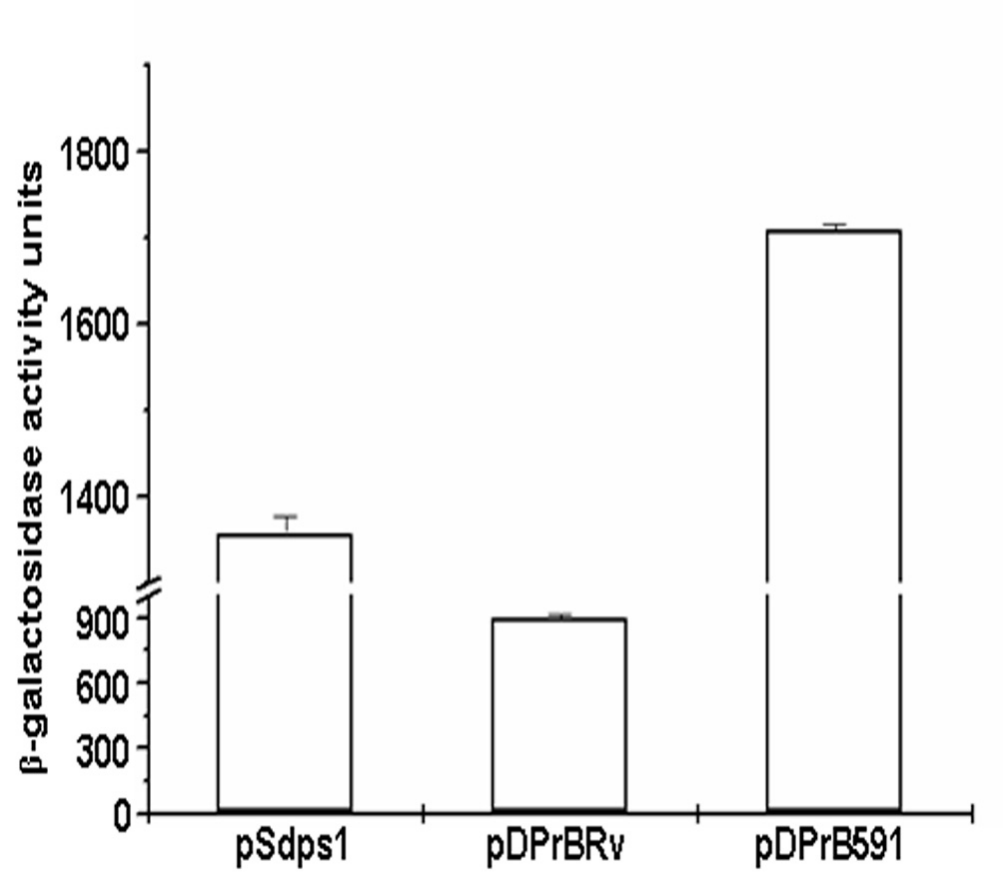
**Regulation of heterologous promoter by IGPr**. *dps *promoter activity under induced conditions in different constructs in terms of *β*-gal activity units expressed as nmol ONPG converted to o-nitrophenol per min per milligram of protein. The transformants were grown in Middlebrook 7H9 medium supplemented with 0.02% glucose (Induced). Each experiment was carried out in triplicates and S.D is indicated as error bars.

To examine if the negative regulatory site, -100 to +1 region of IGPr functions independent of the associated promoter activity, we cloned it downstream of a heterologous promoter in pSdps1, driving the expression of β-galactosidase [23]. pSdps1 has 1 kb upstream region of the gene MSMEG_6467 from *M.smegmatis*. The promoter in pSdps1 is inducible under glucose starvation; at 0.02% glucose in Middlebrook 7H9 liquid medium in stationary phase [23]. By inclusion of +1 to -100 from IGPr of H37Rv (pDPrBRv) the promoter activity decreased by 35% relative to the control plasmid pSdps1 (895 versus 1358 units, Figure 7). When +1 to -100 from VPCI591 was cloned downstream to *dps *promoter (pDPrB591), the repression was reversed and the promoter activity was enhanced by 25% over that of pSdps1 (1709 versus 1358 units). This shows that negative regulation by IGPr functions in the context of a heterologous promoter also.

## Discussion

The *mce1 *operon is different from other three *mce *operons in having Rv0166, a fatty acyl CoA synthetase that catalyzes the initial step in lipid degradation [4,24]. On the other hand, *mce4 *operon is known to be a part of the regulon involved in cholesterol metabolism, however it seems to be just one of the many possible lipid substrates. Furthermore, it is speculated that *mce1 *operon may not have a role in cholesterol import as the loss of Mce1 transporter system does not appear to affect the residual uptake of cholesterol in *mce4*- deficient strain [25].

The presence of 200 base pairs of non-coding sequence between Rv0166 and Rv0167 is yet another feature peculiar to *mce1 *operon among the other four operons present in *M.tuberculosis*. In most other operons and also the other genes within *mce1 *operon, the intergenic distance is not more than one or two codons and often the translation initiation site of one gene is within the coding sequence of the adjacent gene [12]. *In silico *analysis using GeneRunner software shows the absence of any ORFs in the intergenic region between Rv0166 and Rv0167, while ribosomal binding site corresponding to the translational start site of Rv0167 is reported in Tuberculist database. Although most prokaryotes do not have introns, the intergenic region in transcripts serve as substrates for several endonucleases such as RNaseP involved in mRNA processing and hence are implicated in the regulation of gene expression [26-29].

We have characterized the promoter and negative regulatory activity in the surrogate host *M.smegmatis*, but the detection of two active transcription initiation sites both in *M.tuberculosis *H37Rv and VPCI591 suggests both promoters are functional in their native context also.

However the increased promoter strength of the regulatory region from VPCI591 in *M.smegmatis *is not reflected in the difference in the transcript levels for *mce1 *operon genes in VPCI591 as compared to *M.tuberculosis *H37Rv. This may have two reasons, one that both P1 and P2 promoters are active *in vivo *and therefore contribute to the transcript levels in both the strains, while in *M.smegmatis *we observe a clear upregulation of P2 when the negative regulation is lost due to point mutation and P1 is absent (since only P2 is cloned in the plasmid). Further, the difference in fold increase in *β*-galactosidase activity vis-ΰ-vis its transcript levels are significantly different. Similar discordance between protein and mRNA levels is reported in Mycobacteria and *S.cerevisiae *[20-22]. Moreover, *in vivo mce1 *operon could be under the regulatory influence of several factors acting directly or indirectly [4].

We looked for concordance in the expression level of Rv0166 and 0167, as polycistronic mRNA including Rv0166 in *M.tuberculosis *is reported by Casali *et al. *[4]. For comparison, we examined the expression of pairs of adjacent genes in five different operons including Rv1964 and Rv1965 of *mce 3 *operon, Rv2498c and Rv2499c of CitE-scoA operon along with that of Rv0166 and Rv0167 of *mce1 *operon. The expression data was taken from published microarray profiles of *M.tuberculosis *H37Rv cells grown in culture [30]. Pearson's correlation coefficient in the range of 0.8 to 0.58 is observed in all cases except Rv0166 and Rv0167 of *mce1 *operon [0.24; Additional file 2]. Similar difference between coefficient of correlation was observed when we considered the data from clinical isolates grown in Middlebrook 7H9 medium [31]. These results imply that the transcript level is lower for Rv0166 compared to Rv0167, as Rv0166 can be transcribed only from P1 while Rv0167 can be transcribed from both P1 and P2 promoters. Thus lending support to our data suggesting that both promoters of *mce1 *operon are active in cells in culture.

Though *M. tuberculosis *system is replete with examples where the expression of an operon is driven by multiple promoters [32-34], the promoters are known to drive the expression of all the genes of the operon. A study on *furA*-*katG *region shows differential regulation of two *katG *promoters resulting in two different transcripts depending on the stage of infection of *Mycobacterium tuberculosis *[35]. However the consequences of transcription from intergenic promoter could be different. It can only be speculated that two different polycistronic mRNA varying in coding capacity for a catalytic function can be produced by *mce1 *operon: one that includes fatty acyl-CoA synthase (Rv0166) and other lacking it, in absence of *in vivo *infection data. This suggests the possible modulation of the function of *mce1 *operon in cell entry and lipid metabolism vis-ΰ-vis its catalytic function. However, it remains to be examined if the intergenic promoter/regulatory region in *mce1 *operon could bring about differential regulation during infection.

The *mce1 *and *mce2 *operons are known to be negatively regulated by divergently transcribed genes mapping immediately upstream of the operon [4,36]. Though Mce1R, the product of Rv0165c is characterized as a negative regulator of *mce1 *operon, its binding site is not deciphered so far. The results of Casali *et al*. [4] suggest that the site of interaction of Mce1R is in a region upstream of Rv0166, while the negative regulatory element we have identified is downstream to Rv0166. Further we failed to detect direct binding of intergenic promoter with purified His-tagged Rv0165c cloned in pET-28a in gel-shift assays even at high molar ratio of protein to DNA (2000:1). Therefore, it appears that *mce1 *operon has more than one negative regulator. However, it is interesting to note that a heterologous promoter in pSdps1 is also down regulated by the regulatory region of -100 to +1 fragment of IGPr, thus demonstrating that the 100 bp fragment is necessary and sufficient for repressive activity.

Casali *et al*. [4] also observed that *mce1 *operon can be repressed independent of Mce1R by incubation in DMEM medium and suggest that *mce1 *operon may be under multiple negative regulators. Based on their study on lipid degradation operon Kendall *et al. *[24] observed that operon regulation may be more complex than one would expect for a prokaryotic system and may not be guided by just a single regulator.

## Conclusions

Our data strongly supports the presence of two functional promoters for *mce1 *operon in *M.tuberculosis *that could potentially segregate different functions of a single operon. Our results demarcating the regulatory sequences in the intergenic region of *mce1 *operon provide a handle for identifying interacting factors and studying the implications of derepression in the clinical isolate.

## Methods

### *In silico *analysis

The non-coding sequence was detected through ORF analysis of *mce1 *operon using Gene Runner Version 3.01 available at http://www.generunner.net. To identify promoter-like sequences in the intergenic region, the 200 base pair sequence between Rv0166 and Rv0167 was aligned with validated promoter sequences given by Bashyam *et al. *[18]. The presence of a consensus motif was analysed using the MEME program http://meme.nbcr.net/meme3/meme.html available in the public domain [37].

### Enzymes and Chemicals

Restriction enzymes, T4 DNA ligase, RNase free DNaseI were purchased from MBI Fermentas. Kanamycin was from Himedia laboratories Pvt. Ltd., India. The reagents for competent cell preparation, transformation, reporter assays were obtained from Sigma laboratories, USA. [γ-^32 ^P] ATP was from Board of Radiation and Isotope Technology, India.

### Bacterial strains and culture conditions

All the strains and plasmid constructs used in the present study are described in Additional file 3. *M.smegmatis mc*^2^*155 *(ATCC 700084) was obtained from Dr. Anil Tyagi, South Campus, University of Delhi and *Mycobacterium tuberculosis *H37Rv were obtained from Central Jalma Institute for leprosy, Agra, India; *Mycobacterium tuberculosis *VPCI591 is a clinical isolate from Vallabhbhai Patel Chest Institute; Delhi. *M.tuberculosis *strains were grown in Middlebrook 7H9 broth supplemented with OADC (Oleic acid, Bovine albumin fraction V, dextrose-catalase) from Difco laboratories, USA and 0.05% Tween 80 (Sigma). *M.smegmatis *was grown either in Middlebrook 7H9 supplemented with glycerol or on Middlebrook 7H11 plates. Middlebrook 7H9 medium was supplemented with appropriate concentration of glucose whenever *M.smegmatis *clones with *dps *promoter were grown, as specified in the results section. Cloning was carried out in *Escherichia coli DH5α *(Stratagene) grown in Luria-Bertani medium (Difco laboratories, USA). Kanamycin (20 μg/ml) was included for maintenance of plasmids. Transformation in *Escherichia coli DH5α *was carried out using heat shock method [14] and in *M. smegmatis mc*^2^*155 *by electroporation [19] using Gene Pulser (Bio Rad Laboratories Inc. Richmond, California) at 2.5 kV, 25 μF and 1000 Ù in 0.2 cm gap electroporation cuvettes.

The primers used are listed in Additional file 4. The intergenic region of Rv0166-Rv0167 was PCR amplified using primers Mce1AF and Mce1AR from genomic DNA of *Mycobacterium tuberculosis *H37Rv and the clinical isolate VPCI591, cloned in XbaI-SphI sites of pSD5B [Additional file 4, [38]]. Deletion constructs were created by PCR amplification of selected region with specific primers followed by cloning in XbaI-SphI sites of pSD5B. Fragment corresponding to +1 to -100 region of intergenic promoter region (IGPr) was amplified from both *M.tuberculosis *H37Rv and VPCI591 strain, cloned in the vector pSdps1 downstream of glucose regulated *dps *promoter [23,39] to generate pDPrBRv and pDPrB591 respectively at VspI-PstI site and electroporated into *M. smegmatis mc*^2^*155*. pSdps1 has 1 kb upstream region of *dps *gene (MSMEG_6467, DNA binding protein from starved cells) from *M. smegmatis*. The transformants were screened by PCR, confirmed by restriction digestion and sequencing.

The expression of *β*-galactosidase was assayed both in the log (O.D._600 _0.8) and stationary phase (O.D._600 _2.0) cultures of the transformants using modified protocol of Miller *et al. *[40]. Promoter activity is expressed as units of *β*-galactosidase activity in terms of ONPG converted to o-nitrophenol (nmol per min per mg of protein).

### Mapping transcription start site

The transcription start site was mapped using the strategy described by Lloyd *et al. *[41]. Primer extension was carried out on DNA free RNA with fluorescence labeled primers HEX-tsp1 and FAM-tsp2 mapping 100 nucleotides downstream of the translation initiation site of Rv0166 and Rv0167 respectively [Additional file 4]. The DNA sequence analysis and Genescan analysis was carried out at the commercial facility of The Centre for Genomic Application, Okhla, New Delhi and Labindia, Udyog Vihar, Gurgaon, India respectively. The Genescan analysis was carried out on 3130×l Genetic Analyzer from Applied Biosystems with GSLIZ 500 as marker set. The data was analyzed using GeneMapper V4.0.

### Quantitative RT-PCR

The transcriptional activity in log and stationary phase, was estimated by quantitative PCR using cDNA samples. 15 ml cultures of *M.tuberculosis *H37Rv and VPCI591 from log (day10) and stationary phase (day 20) were harvested at 4°C. RNA isolation was performed using RNeasy Mini Kit (Qiagen) and treated with DNaseI (MBI Fermentas). Absence of amplicons in PCR without reverse transcriptase confirmed the absence of DNA contamination. 500 ng of DNase I treated total RNA samples extracted were retrotranscribed using cDNA synthesis kit (MBI Fermentas) with random hexamer primers. Real Time PCR was performed using SYBR Green PCR master mix (Applied Biosystems, USA); *sigA *or rpoB was used as endogenous control. The relative expression of *mce1 *operon genes (Rv0167, Rv0170 and Rv0178) in *M.tuberculosis *H37Rv and VPCI591 and *lacZ *expression from the clones pPrRv and pPr591 in *M.smegmatis *was determined, using similar protocol. The experiments were repeated three times and the data was analyzed using the ΔΔCt method [42].

## Authors' contributions

VB and MB conceived the project. RP helped with M.tuberculosis culturing. SB and MJ contributed equally to the experiments. VB, MB, SB and MJ participated in experiment design and data interpretation and manuscript preparation. All authors read and approved the manuscript.

## Supplementary Material

Additional file 1 **Detection of putative promoter motif**. Output consensus sequences of MEME mapped [bold upper case] on validated promoter sequences. The input sequences are from T6 to PA [gyr]. IGPr is the query sequence. Translation start site (ATG/GTG) of the gene driven by each promoter used as the reference for alignment is shown in capital. Click here for file

Additional file 2 **Comparison of expression level of adjacent genes in different operons**. Pearsons correlation coefficient of the first two genes of *mce1 *operon is compared to that of neighbouring genes in five different operons. Operons predicted by Roback *et al *[43] and Moreno-Hagelseib *et al *[44] used; * represents the operons extending from Rv1460 to Rv1466 (operon A) and Rv3083-3089 (operon B). Least correlation is observed between Rv0166 and Rv0167. Expression data of Fu and Fu-Liu [30] was taken for analysis. Click here for file

Additional file 3 Strains and plasmids used in the present study. Click here for file

Additional file 4 List of primers. Click here for file
