# Supplementary material for: Functional analysis of an intergenic non-coding sequence within mce1 operon of M.tuberculosis
Source: BMC Microbiol. 2010 Apr 27;10:128. doi: 10.1186/1471-2180-10-128 (PMC2867952; doi:10.1186/1471-2180-10-128)
Supplement: Additional file 1 — Detection of putative promoter motif. Output consensus sequences of MEME mapped [bold upper case] on validated promoter sequences. The input sequences are from T6 to PA [gyr]. IGPr is the query sequence. Translation start site (ATG/GTG) of the gene driven by each promoter used as the reference for alignment is shown in capital. [file 1471-2180-10-128-S1.DOC]

>T6

gcaggaacggcaagaacaaaacgaaactctccatcaacccgtctttcgtattggtattgcgatcacggtgccaggcctacccgcgggccgcgcacctggtaacagtccagtgtgcccgtccagtctggcaggccggaaacatcggtcagcagataggct**ttaccagc**gatgtgaaccggcgagccgggtgaggaggatctGTG

>T26

tcatcgacgggaggcacccggctggctccgttgtcactcatggtccaacatcctgccgcatcaccaccgcacgcggcatatgatgctcgcagtcgcggtggtgcggcc**ttatcgcc**atgagcgaaatcttctgtatcactgatcattccgagcctatgacggccc**GGTTCTT**gtcagtggtgcttcgtagaatccgaggcATG

>T101

ggcgcgtaccagcgaatggcacagccaccgcagtcgccgacatcccgcgaagatgtggcagattttcgtgcggtcgagccggcgaaggcctagcgtca**ttgttgcc**tggcaa**GGTTGCT**gggcccggcgatcgcagccgacgtgatacctgaccgttgttgatagtgtcggcggcagcgggttggttgggaggatcagcgATG

>T119

acgaaggccgccgcctcggcagcaagg**ttttcggc**cacagagcgcagccgcgcg**ggttcgt**tgtcaggtcgtgtcaccggcctatcgcatcacagtcgccacccgcatggtggcgtggactccagcggccataacgccctcgcaactgccgggccgcagtttaaggtgagggtcatccacgtctcgccgaggagattcgaATG

>T125

gaccacaggctggtcgaagcggctctggatcgatgactatagatgaccgagcggcagcctgtacgattaaccgaaatt**ttttgacct**cgccgtaaacgatgtaaacggagtgtacaaaactgccccaacgagtggcgtctccccggtag**GGTTCGT**cctcacgcggcggcttgttgaagcgatggaatcaggaaggtaaaATG

>T129

gtcgcgtcgcaggattcacactcggagcatgagccggcgcgccgcgatcggcagtcgggtgcaagcaagtcggccgactcgcgggcagga**ttaccgcc**cgac**GGTTCCT**ggcgtggttcaatattcgccgaagaagcgcctacgtaggccaagtcattcgtacacattgagaattcgccggaagggcccaggggaaagcgATG

>T130

aaagcgcgtgcgggtgagatcacgcacttcaccgggatcgacagcccatatcagcggcccaagaacccagacctacggcttacgccggatcgcagcatagacgagcaggcgcaggagg**ttatcgac**ctgttggagtcatcgtcttaggccggcct**GGTTGCT**ctgctgtccctggcaagcgggtggcacaatcctgaagcATG

>T150

caggcgctcccgtcgttccaggagggcgattgtcttgtcggggaactcggccgtcacgtgacaggccgtcccgatgccggataggccggcgccgatgatcaggacgtcgaaatgct**GGTTCAT**ggctccagcatggtggaggacccccgccacgtattgacac**tttgcgac**agccttttatcattttccgacaggaggtgATG

>PA(gyr)

gtgggagaccaccatggatccctcggttcgtgtgttgcgtcaagtgacgctggacgacgccgccgccgccgacgagttgttctccatcctgatgggcgaggacgtcgacgcgcggcgcagct**ttatcacc**ccgccaacgccaaggatgttc**GGTTCCT**ggatgtctaacgcaaccctgcgttcgattgcaaacgaggaatagATG

**>IGPr**

ttgcccg**CTATTGAC**gaagggttaaatgtgcggatgccttacactcctggctggccatcgggta**GATTCCT**gtggtctccgttactccctgtgagtaacgaggtggcggtcacacaccaagggtcggggcaaggaggagcgtgcgacatgatgcgccgcggcgccgcgatacccaggtcggcggcttgagggagccgcgATG

**Additional File 1**

Consensus sequences detected and mapped by MEME (bold upper case) on validated promoter sequences. The input sequences are from T6 to PA(gyr). IGPr is the query sequence. Translation start site (ATG/GTG) of the gene driven by each promoter used as the reference for alignment is shown in capital.
