# Supplementary material for: Functional analysis of an intergenic non-coding sequence within mce1 operon of M.tuberculosis
Source: BMC Microbiol. 2010 Apr 27;10:128. doi: 10.1186/1471-2180-10-128 (PMC2867952; doi:10.1186/1471-2180-10-128)
Supplement: Additional file 2 — Comparison of expression level of adjacent genes in different operons. Pearsons correlation coefficient of the first two genes of mce1 operon is compared to that of neighbouring genes in five different operons. Operons predicted by Roback et al [43] and Moreno-Hagelseib et al [44] used; * represents the operons extending from Rv1460 to Rv1466 (operon A) and Rv3083-3089 (operon B). Least correlation is observed between Rv0166 and Rv0167. Expression data of Fu and Fu-Liu [30] was taken for analysis. [file 1471-2180-10-128-S2.DOC]

| **Operon** | **I^st^ gene** | **II^nd^ gene** | **Pearsons correlation coefficient** |
| --- | --- | --- | --- |
| mce1 | Rv0166 | Rv0167 | 0.24 |
| mce3 | Rv1964 | Rv1965 | 0.66 |
| CitE-scoA | Rv2498c | Rv2499c | 0.78 |
| argC-argG | Rv1652 | Rv1653 | 0.58 |
| Operon A* | Rv1460 | Rv1461 | 0.89 |
| Operon B* | Rv3083 | Rv3084 | 0.88 |

**Additional file 2**. Comparison of expression level of adjacent genes in different operons. Pearsons correlation coefficient of the first two genes of *mce1* operon is compared to that of neighbouring genes in five different operons. Operons predicted by Roback *et al* [43] and Moreno-Hagelseib *et al* [44] used; * represents the operons extending from Rv1460 to Rv1466 (operon A) and Rv 3083-3089 (operon B). Least correlation is observed between Rv0166 and Rv0167. Expression data of Fu and Fu-Liu [30] was taken for analysis.
