# Supplementary material for: Functional analysis of an intergenic non-coding sequence within mce1 operon of M.tuberculosis
Source: BMC Microbiol. 2010 Apr 27;10:128. doi: 10.1186/1471-2180-10-128 (PMC2867952; doi:10.1186/1471-2180-10-128)
Supplement: Additional file 3 — Strains and plasmids used in the present study. [file 1471-2180-10-128-S3.DOC]

| **No.** | **Strain/Plasmid** | **Description** | **Source** |
| --- | --- | --- | --- |
| 3 | *M. tuberculosis* H37Rv | Standard lab strain of *Mycobacterium tuberculosis.* | Central Jalma Institute for leprosy, Agra, India |
| 4 | VPCI591 | MDR Clinical isolate of *Mycobacterium tuberculosis.* | Vallabhbhai Patel Chest Institute, Delhi. |
| 5. | pSD5B | Mycobateria-*E.coli* shuttle vector with promoterless LacZ as reporter. | Dr.Anil Tyagi |
| 11 | pSdps1 | *dps* gene promoter from *M.smegmatis* cloned upstream of *LacZ* in pSD5B. | Dr. Dipankar Chatterji |
| 14 | pSD5WP | A promoter clone from the mycobacterial promoter library in pSD5B characterized with low levels of promoter activity. | Dr.Anil Tyagi |
| 15 | pSD5SP | A promoter clone from the mycobacterial promoter library in pSD5B characterized with high levels of promoter activity. | Dr.Anil Tyagi |

**Additional File 3** Strains and plasmids used in the present study.
