# Supplementary material for: Functional analysis of an intergenic non-coding sequence within mce1 operon of M.tuberculosis
Source: BMC Microbiol. 2010 Apr 27;10:128. doi: 10.1186/1471-2180-10-128 (PMC2867952; doi:10.1186/1471-2180-10-128)
Supplement: Additional file 4 — List of primers. [file 1471-2180-10-128-S4.DOC]

| **No.** | **FP** | **Sequence** | **RP** | **Sequence** | **Construct** |
| --- | --- | --- | --- | --- | --- |
| 1. | Mce1AF | 5’ATTGTATCTAGAGCCCGCTATTGA CG 3’ | Mce1AR | 5’GTCGTCGCATGCGCTCCCTCAAGCCG 3’ | pPrRv,  pPr591 |
| 2. | Mce1PBFP | 5’AGGAGGCTCTAGACATGA TG 3’ | Mce1AR | 5’GTCGTCGCATGCGCTCCCTCAAGCCG 3’ | pPrA |
| 3. | Mce1PCFP | 5’TAACGATCTAGAGGTCAC AC3’ | Mce1AR | 5’GTCGTCGCATGCGCTCCCTCAAGCCG 3’ | pPrB591 |
| 4. | Mce1PEF | 5’GGCCATCGTCTAGATTCC TG 3’ | Mce1P5R | 5’ATCATGTCGCATGCCTCCTC 3’ | pPrC |
| 5. | Mce1PAFP | 5’ACGTTTCTAGACTATTGACG 3’ | Mce1P2RP | 5’CCACCTGCATGCTCACAG GGAG 3’ | pPrD |
| 6. | NT1-100F | 5’TAACGAATTAATGGTCAC AC 3’ | NT1-100R | 5’GTCGTCCTGCAGGCTCCCTCAAGCCG 3’ | pDPrBRv  pSPrB591 |
| 7. | rpoBRTF | 5’CTTCGACGAGACCATTGAC 3’ | rpoBRTR | 5’CGCTTGTCGACGTCAAAC 3’ | *rpoB* |
| 8. | Rv0167F | 5’ACAACGCTTGGCGGTTAC 3’ | Rv0167R | 5’AAACAGCGCCTTTCCAGTC 3’ | Rv0167 |
| 9. | Rv0170F | 5’GGAACCGTCGTCAAACTC 3’ | Rv0170R | 5’CATTAGTCCGGTCGAAGC 3’ | Rv0170 |
| 10. | Rv0174F | 5’CCAGTGCTGACTCGCTTCATC 3’ | Rv0174R | 5’TCGGAATTCGCAAGGTAG TACC 3’ | Rv0174 |
| 11. | MysAF | 5’CGTTCCTCGACCTCATCCA 3’ | MysAR | 5’TCGAGAACTTGTAGCCCTTGGT3’ | *sigA* |
| 12. | lacZF | 5’CCCGCATTGACCCTAACG 3’ | lacZR | 5’TCAGCACCGCATCAGCAA 3’ | *lacZ* |
| 7. | FAM-tsp2 | 5’TTCCAGTCAGCACACACA TG 3’ |  |  | primer extension |
| 8. | HEX-tsp1 | 5’GTTCGAGCTGGTTGACCC 3’ |  |  | primer extension |

**Additional File 4** Primers used in the present study.
